# Endoscope‐Assisted Nipple‐Sparing Mastectomy With Immediate Implant Reconstruction: A Retrospective Study

**DOI:** 10.1002/wjs.70316

**Published:** 2026-03-20

**Authors:** Yuan Shao, Youcheng Zhang, Huafeng Kang, Qing Zhou, Ziliang Zhang, Guiru Yan, Binghua Kan

**Affiliations:** ^1^ Department of Surgical Oncology Hanzhong Central Hospital Hanzhong Shaanxi China; ^2^ General Surgery Department Second Hospital of Lanzhou University Lanzhou Gansu China; ^3^ Breast Disease Diagnosis and Treatment Center Second Affiliated Hospital of Xi'an Jiaotong University Xi'an Shaanxi China

**Keywords:** breast cancer, endoscopic surgery, implant‐based breast reconstruction, nipple‐sparing mastectomy

## Abstract

**Background:**

Breast cancer surgery increasingly favors nipple‐sparing and endoscopic techniques to optimize oncological safety, cosmetic outcomes, and patient satisfaction. We aimed to evaluate and compare the clinical efficacy and postoperative safety profiles of endoscope‐assisted and conventional open approaches for nipple‐sparing mastectomy with immediate prosthetic reconstruction in breast carcinoma management.

**Methods:**

This retrospective study evaluated 75 consecutive patients undergoing nipple‐sparing mastectomy with concurrent prosthetic reconstruction at a tertiary referral center (Hanzhong Central Hospital) between December 2021 and December 2023. Patients were allocated into two groups based on the surgical modality: endoscope‐assisted (*n* = 35) versus conventional open (*n* = 40) approaches based primarily on patient preference and secondarily on surgeon selection. In cases of significant comorbidities, conventional open mastectomy (or state type) was performed.

**Results:**

We observed significant differences in procedural characteristics between the endoscope‐assisted and conventional open surgery cohorts. The endoscopic cohort had a 23.5% prolongation in operative duration, with a 64% reduction in total incision length. No significant differences were observed between the groups in critical clinical outcomes, including intraoperative hemostasis parameters, postoperative drainage volumes, complication profiles, or oncological safety markers. Implant removal was required in one patient in each group. Patient‐reported outcome measures showed equivalence in BREAST‐Q domains assessing chest wall integrity, psychosocial adaptation, and sexual well‐being, along with comparable Harris functional assessment scores. The endoscopic approach yielded superior scar cosmesis as quantified using the SCAR‐Q evaluation.

**Conclusions:**

Endoscope‐assisted nipple‐sparing mastectomy with immediate prosthetic reconstruction is not inferior to conventional open techniques regarding perioperative safety, oncological radicality, and multidimensional patient satisfaction metrics. This approach provides enhanced aesthetic outcomes through minimized cutaneous trauma.

## Graphical abstract

Breast cancer surgery increasingly favors nipple‐sparing and endoscopic techniques to optimize oncological safety, cosmetic outcomes, and patient satisfaction. We aimed to evaluate and compare the clinical efficacy and postoperative safety profiles of endoscope‐assisted and conventional open approaches for nipple‐sparing mastectomy with immediate prosthetic reconstruction in breast carcinoma management. This retrospective study evaluated 75 consecutive patients undergoing nipple‐sparing mastectomy with concurrent prosthetic reconstruction at a tertiary referral center (Hanzhong Central Hospital) between December 2021 and December 2023.

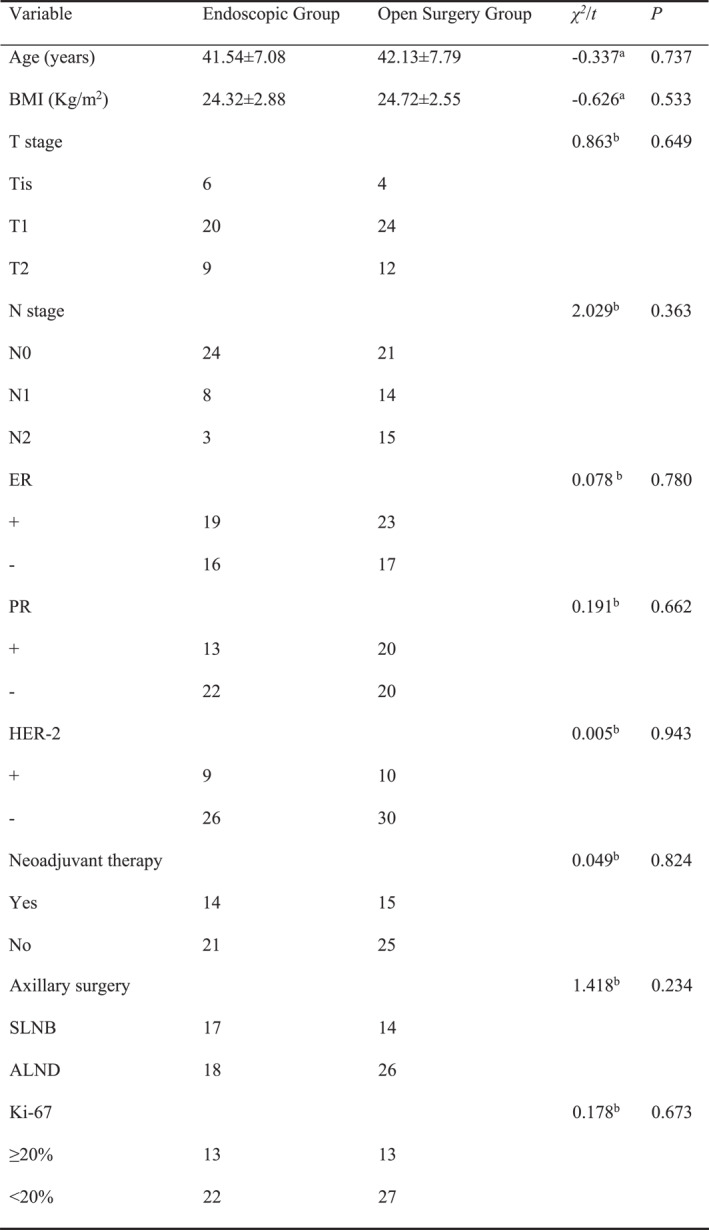

## 1 Introduction

Breast cancer is reportedly the most common cancer in women, exhibiting the highest age‐standardized incidence and mortality rates among cancers [1]. Contemporary breast cancer surgery emphasizes organ preservation, with breast‐conserving therapy chosen by 62.7% of new patients (National Cancer Database). Oncoplastic surgery, notably nipple‐sparing mastectomy with immediate reconstruction, addresses both oncological safety and cosmetic outcomes by preserving breast contour, resulting in high patient satisfaction [2]. Conventional open nipple‐sparing mastectomy (NSM) disrupts the skin flap vascular network, increasing the risks of infection, necrosis, and implant loss—particularly with prepectoral implants—while scarring may compromise aesthetic outcomes [3]. Endoscopic breast reconstruction is increasingly adopted after mastectomy, demonstrating superior outcomes over open surgery [4]. Technical refinements, including single‐port insufflation NSM, enhance oncological resection via 10‐mm ports with 8‐mmHg CO_2_, thereby optimizing visualization while preserving nipple–areolar viability. We retrospectively analyzed these prospectively maintained data regarding subpectoral breast reconstructions to evaluate critical end points, such as patient‐reported outcomes and objective aesthetic evaluation.

## 2 Material and Methods

### 2.1 General Information

This single‐center observational study involved analysis of consecutive patients diagnosed with breast cancer and treated with NSM and immediate implant‐based reconstruction at the Department of Surgical Oncology in our hospital between December 2021 and December 2023. Analyses included 75 women (age: 25–58 years and mean: 41.85 ± 7.42). Patients were divided into two groups based on surgical approach: (1) endoscopic (*n* = 35), aged 25–58 years (mean: 41.54 ± 7.08) and (2) open surgery (*n* = 40), aged 26–56 years (mean: 42.13 ± 7.79). Surgeries were performed through axillary incisions in the endoscopic group. In the open surgery group, surgical incisions included inframammary fold incisions in 18 , radial incisions in 15, and periareolar incisions in seven cases. Notably, 18 and 26 patients in the endoscopic and open surgery groups, respectively, underwent axillary lymph node dissection and received postoperative radiotherapy. No patient underwent implant reconstruction with mesh assistance. Demographic homogeneity (body mass index [BMI]/Stage/Ki67) was confirmed between the cohorts through standardized baseline parameter analysis (Table 1; all comparative *p* > 0.05). The study protocol was approved by the Institutional Review Board of Hanzhong Central Hospital (IRB#202424). The study was complied with the principles of the Declaration of Helsinki. Written informed consent was obtained before surgical intervention through standardized disclosure processes.

**TABLE 1:** Comparison of baseline characteristics between the two groups (*n* = 75).

Variable	Endoscopic group	Open surgery group	*χ* ^*2*^/*t*	*p*
Age (years)	41.54 ± 7.08	42.13 ± 7.79	−0.337^a^	0.737
BMI (kg/m^2^)	24.32 ± 2.88	24.72 ± 2.55	−0.626^a^	0.533
T stage			0.863^b^	0.649
Tis	6	4		
T1	20	24		
T2	9	12		
N stage			2.029^b^	0.363
N0	24	21		
N1	8	14		
N2	3	15		
ER			0.078^b^	0.780
+	19	23		
−	16	17		
PR			0.191^b^	0.662
+	13	20		
−	22	20		
HER‐2			0.005^b^	0.943
+	9	10		
−	26	30		
Neoadjuvant therapy			0.049^b^	0.824
Yes	14	15		
No	21	25		
Axillary surgery			1.418^b^	0.234
SLNB	17	14		
ALND	18	26		
Ki‐67			0.178^b^	0.673
≥ 20%	13	13		
< 20%	22	27		

*Note:* T and N stages are based on postoperative pathological staging. ER, PR, and HER‐2 positivity were determined as at least one positive result from biopsy pathology or postoperative pathology. HER‐2 (2+) cases were confirmed as positive by fluorescence in situ hybridization testing.

Abbreviations: ALND, axillary lymph node dissection, including either axillary dissection following a positive sentinel lymph node biopsy or direct axillary dissection for clinically positive axillary lymph nodes before neoadjuvant therapy; BMI, body mass index; ER, estrogen receptor; HER‐2, human epidermal growth factor receptor 2; PR, progesterone receptor; SLNB, sentinel lymph node biopsy.

^a^ *t*‐test.

^b^ Chi‐square test.

### 2.2 Surgical Methods

The endoscopic and open surgery groups required preoperative breast marking. All surgical procedures were performed by a consistent surgical team. The selection of the operative technique (endoscopic vs. open) was guided by patient's preference, with the patient's physical condition being a secondary consideration. For instance, if a patient had severe cardiopulmonary disease and could not tolerate pneumoperitoneum or prolonged anesthesia, open surgery was recommended. Factors such as tumor size and location were not considered reference criteria for selecting the surgical approach. Patients stood upright, and the breast was gently displaced cephalad (superior), medially, and laterally to outline the superior, medial, and lateral boundaries, respectively. The inferior boundary was demarcated along the inframammary fold. The anterior axillary line was traced with the arm in the neutral dependent position. Axillary lymph node dissection was performed directly for patients requiring neoadjuvant therapy and confirmed to have axillary lymph node positivity through core needle biopsy. In other patients, a suspension of nanocarbon was injected into the periareolar skin to trace sentinel lymph nodes. A sentinel lymph node biopsy was performed, and the nodes were sent for frozen pathological examination. The decision to perform axillary lymph node dissection was based on lymph node assessment results. Single‐stage immediate reconstruction with silicone implants was performed. Prepectoral implant placement was chosen for patients who did not require postoperative radiotherapy. Subpectoral implant placement was selected for patients who required postoperative radiotherapy. Implants were provided by a Shanghai‐based company specializing in medical devices for cosmetic surgery. The initial anti‐infection regimen for the first approximately 40 patients was intravenous cefuroxime (1.5 g) 30–60 min preoperatively, with an additional 1.5‐g dose administered if surgery exceeded 180 min, followed by 1.5 g every 8 h for 24 h postoperatively. The regimen was subsequently optimized to include sequential oral therapy with cefuroxime axetil (0.25 g twice daily for 7 days) after the initial 24‐h intravenous course. Regarding total incision length, in the endoscopic group, the procedure was performed through a single transaxillary incision; in the open group, the total incision length was defined as the sum of the breast incision and axillary incision lengths. For further details, please refer to Supporting Information S1: Methods and Figures S1–S6.

### 2.3 Statistical Methods

Statistical analyses were conducted using the Statistical Package for Social Sciences (version 26.0; IBM Corp., Armonk, NY, USA) according to predefined analytic protocols. Continuous variables demonstrating parametric distribution (Shapiro–Wilk *p* > 0.05) were presented as arithmetic mean ± standard deviation, with between‐group differences assessed via independent samples t‐tests incorporating Levene's equality of variance verification. Nonnormally distributed metrics were subjected to Box–Cox transformation; persistent nonparametric characteristics were reported as median (interquartile range), with Mann–Whitney U tests for comparative analysis. Categorical variables were presented as frequency distributions and analyzed using *χ* ^2^ tests with Yates' continuity correction, supplemented by Fisher's exact test with cell counts < 5. All inferential statistics incorporated 95% confidence intervals (CIs), with the α‐level set at 0.05 (two‐tailed). Effect size estimations included Cohen's *d* for parametric comparisons and phi coefficients for contingency tables, aligning with the American Psychological Association (7th edition) reporting standards.

### 2.4 Generative AI in Scientific Writing

AI‐assisted manuscript refinement (DeepSeek; https://www.deepseek.com) was used in this document. Language polishing was performed using DeepSeek's NLP tool kit.

## 3 Results

### 3.1 Perioperative Indicators

The endoscopic group had higher intraoperative blood loss (34.57 ± 10.03 mL) and total drainage volume (294.00 ± 83.78 mL) than did the open surgery group (30.63 ± 11.45 and 277.25 ± 67.79 mL, respectively), with no significant difference (*p* > 0.05). The operative time in the endoscopic group (198.71 ± 31.82 min) was significantly longer than that in the open surgery group (123.50 ± 17.77 min), whereas the total incision length in the endoscopic group (6.07 ± 2.18 cm) was significantly shorter than that in the open surgery group (14.80 ± 1.26 cm) (*p* < 0.001) [Supporting Information S1: Table S1].

### 3.2 Postoperative Complications

Each group had two cases of transient nipple–areola complex ischemia. The blood supply recovered fully and returned to normal after close observation. In the open surgery group, one patient required excision of the nipple–areola complex because of cancerous tissue invasion. In the endoscopic group, one patient developed local skin necrosis on postoperative Day 2. The affected area was approximately 3 × 3 cm. The bandage was immediately loosened to avoid breast compression, and antibiotics were administered to prevent infection. After plastic surgery consultation, the necrotic area increased to 5 × 4 cm within 6 h. Consequently, the implant was removed on postoperative day 3. Another patient in the endoscopic group experienced necrosis in a small skin area (≈1 × 1 cm) near the axillary tail of Spence. The necrosis, caused by pressure at the endoscopic puncture site, healed spontaneously with conservative management involving enhanced dressing changes. Two patients in the laparoscopic group and three in the open group experienced incision infections. For two of the infection cases, one from each group, the infection was attributed to a complicated postoperative recovery process. All affected patients recovered after active dressing changes and adequate antibiotic treatment.

No significant differences were observed between groups in incision infection (CDC Grade I), transient nipple–areolar complex ischemia (CDC Grade I), implant infection (CDC Grade II), skin necrosis (CDC Grade IIIa), and implant removal (CDC Grade IIIb) (*p* > 0.05; Supporting Information S1: Table S2).

### 3.3 Comparison of BREAST‐Q, SCAR‐Q, and Harris Scale Scores

The endoscopic group performed better than the open surgery group regarding BREAST‐Q (chest health, psychosocial health, and sexual health) and Harris scores, with no significant differences (*p* > 0.05). Conversely, the SCAR‐Q score was significantly better in the endoscopic group than in the open surgery group (*p* < 0.01; Supporting Information S1: Table S3).

### 3.4 Follow‐Up Results

The postoperative follow‐up period was 6–30 months. Median follow‐up times for the endoscopic and open surgery groups were 16 and 19 months, respectively. In the endoscopic group, one patient had isolated bone metastasis 23 months postoperatively, one had brain metastasis 15 months postoperatively, and another experienced isolated chest wall recurrence. In the control group, each patient had isolated bone metastasis 25 months postoperatively, liver and lung metastases 9 months postoperatively, distant metastasis and local recurrence 16 months postoperatively, and areolar skin recurrence 10 months postoperatively, respectively. No deaths occurred in either group.

The local recurrence rate did not differ significantly between the endoscopic (2.86%) and open surgery (5.00%) groups (*p* > 0.05), with the recurrences distributed as one chest wall recurrence in the endoscopic group and one areolar recurrence and one axillary lymph node recurrence in the open group (Supporting Information S1: Table S2).

Cancer recurrence occurred in one patient in the endoscopic group 3 months following endoscope‐assisted NSM (E‐NSM) with a prepectoral implant, with initial cT2N0 3.6‐cm ER/PR/HER2‐negative invasive ductal carcinoma (IDC) after neoadjuvant chemotherapy with the TAC regimen (docetaxel, doxorubicin, and cyclophosphamide), achieving a pathological complete response; moreover, the pectoral fascia was preserved at mastectomy. Chest wall recurrence of the cancer was detected as a 1.8‐cm mass located at the original cancer site, anterior to the implant. In the open mastectomy group, one case of nipple–areola complex recurrence occurred 5 months postoperatively as triple‐negative ductal carcinoma in situ (DCIS) following open mastectomy with radial incision for DCIS with intraoperative frozen section analysis of the nipple–areola complex margin negativity, without adjuvant chemotherapy. One axillary recurrence (lymph node) occurred 23 months after open mastectomy for the initial cT1N0 1.6‐cm IDC hormone receptor‐negative, HER2‐positive mass with negative sentinel lymph node biopsy results, undergoing postoperative adjuvant therapy with trastuzumab plus paclitaxel.

## 4 Discussion

With advancing technology, patients with breast cancer can receive timely diagnosis and treatment in the early disease stages [5]. The average age of breast cancer onset is higher in Western countries but lower among younger Chinese women [6]. Breast reconstruction, an option for patients with breast cancer who are not candidates for breast conservation, is conducted using either endoscopic or traditional open surgeries, with the former including suspension, insufflation, and lipolysis techniques [7]. However, the suspension technique presents challenges, such as difficulty in establishing breast cavities and a lack of natural appearance. Consequently, the “chopstick effect” may occur [8]. Although the lipolysis technique addresses some issues, it is less effective in patients with minimal subcutaneous breast fat and incurs additional surgical costs [9].

We compared the aesthetic and safety outcomes of E‐NSM with immediate implant insertion and traditional open NSM with immediate implant insertion. Both groups completed the surgeries successfully; however, the endoscopic group had a significantly longer operative time and shorter total incision length than did the open surgery group. These were our first attempts at performing E‐NSM procedures. We encountered challenges such as unfamiliarity with the technique and lack of team coordination, which contributed to prolonged operative times. The total incision length in our endoscopic group was 6.07 ± 2.18 cm, longer than the 3.7 ± 0.7 cm reported by Liu et al. [10]. As the endoscopic technique has not been fully adopted, axillary lymph node dissection often requires extension of the axillary incision up to 10 cm, thereby increasing total incision length.

No significant difference was observed between the groups in intraoperative blood loss and total drainage volume; however, mean values were slightly higher in the endoscopic group than in the open surgery group. These results are due to the team being in the early stages of learning and mastering the endoscopic surgical technique. The medical team actively communicated E‐NSM advantages, leading patients who were initially hesitant about breast reconstruction to select E‐NSM. Consequently, > 60% of the patients with breast cancer and three with gynecomastia chose this method. These patients achieved better cosmetic results and higher levels of satisfaction than those who underwent traditional surgery, thereby corroborating the findings of a previous study [11] and allowing us to become proficient in this technique; in more recent surgeries, operative time decreased by approximately 30 min/surgery.

Regarding surgical technique, we used retrograde dissection for all E‐NSM, which was initiated with the development of the retropectoral space, followed by subcutaneous layer dissection. These steps create sufficient operating space, improve anatomical pathways, and prevent instrument interference, enabling smooth execution of endoscopic surgery [12, 13, 14]. During the surgery, we did not use “Huaxi Hole 1 and 2.” Instead, a small 5‐mm incision was made along the anterior axillary line at the nipple level. A 5‐mm trocar was inserted through the incision, enabling placement of the dissecting forceps, thereby reducing instrument interference. Postoperatively, the incision served as an insertion point for the drainage tube, reducing the need for “Huaxi Holes” without increasing breast trauma.

The differences in blood loss and drainage volume between the endoscopic and conventional surgery groups were not significant. Blood lost in E‐NSM surgery is 24.8 ± 20.0 mL, significantly lower than 39.7 ± 29.9 mL observed in traditional surgery [10]. However, a prospective study reported a blood loss of 25.5 ± 16.0 mL in E‐NSM [3], which was lower than our result. Therefore, our findings showed that endoscopic surgery was not superior regarding blood loss and trauma, possibly due to the limited experience of our team in performing E‐NSM.

We found no significant differences in perioperative complications and oncological safety between groups. The endoscopic group had two cases of skin necrosis: one case occurred near the axillary tail of Spence and was resolved fully with dressing changes, whereas the other developed a secondary infection due to ischemic skin necrosis, leading to implant removal. In this study, open surgery, rather than endoscopic surgery, was selected for managing patients with tumors measuring > 5 cm or those located adjacent to the skin or pectoral muscle. The decision was primarily based on the following considerations: open surgery provides more adequate surgical field exposure, facilitates assessment of the relationship between tumor and adjacent tissues, and aims to achieve complete resection. To maximize oncological safety, standardized intraoperative sampling and pathological examination of the superficial and deep tumor margins were routinely performed. Each group had two cases of transient nipple–areola complex ischemia. The ischemic areas healed with scabbing using enhanced dressing changes and close observation. No complete ischemic necrosis of the nipple–areola complex occurred, and surgical excision was not required [15]. The endoscopic and open surgery group surgeries followed the same steps, aseptic principles, and tumor‐free principles, suggesting that the oncological outcomes are similar [16]. No deaths occurred during the follow‐up period. Local and distant recurrence rates in the endoscopic group were lower than those in the open surgery group. The lower recurrence rates in the endoscopic group may be related to the small sample size and the short follow‐up period.

NSM oncological integrity has been extensively validated using population‐level evidence. Fu et al. demonstrated noninferiority in survival outcomes between NSM and conventional mastectomy cohorts [17]. Contemporary comparative effectiveness research encompassing E‐NSM and robotic‐assisted and conventional NSM approaches reveals equivalent long‐term oncological outcomes, including 5‐year locoregional recurrence‐free rates, disease‐free survival, and overall survival after propensity score matching [18]. These consolidated findings position E‐NSM as meeting the Level 1A evidence criteria for oncological safety, as per the American Society of Clinical Oncology Clinical Practice Guidelines.

E‐NSM has better BREAST‐Q scores, particularly in breast satisfaction, chest physical health, psychosocial health, and sexual health, than traditional NSM [3, 10, 11, 12, 13, 14]. However, in this study, the Breast‐Q scores were higher in the endoscopic group than in the open surgery group. These differences were not significant. SCAR‐Q scores were significantly higher in the endoscopic group than in the open surgery group, suggesting that optimization of E‐NSM techniques can improve outcomes.

Notably, 14 (40%) and 15 (37.5%) patients in the endoscopic and open surgery groups received neoadjuvant therapy, respectively. Neoadjuvant therapy ensures safety and good cosmetic outcomes of E‐NSM and immediate implant placement [19]. No mesh was used in either group. Meshes may improve BREAST‐Q scores and reduce surgical complications, particularly capsular contracture [20], while enhancing breast aesthetics and patient satisfaction [21]. The use of mesh does not significantly affect postoperative outcomes and complications. Prepectoral implant breast reconstruction can improve satisfaction with the breast shape [22].

During postoperative follow‐up, five and three patients in the open surgery and endoscopic groups, respectively, developed significant breast skin wrinkling, poor implant palpability, and implant rippling after a > 10% weight loss. These patients had prepectoral implants and minimal subcutaneous breast fat. Using meshes may increase breast thickness and improve outcomes in similar patients. In the endoscopic group, one patient required implant removal because of skin necrosis; this case also involved prepectoral implant placement. Using mesh during surgery may alleviate skin flap pressure, increase breast thickness, and prevent extensive skin necrosis [23]. In subpectoral implant reconstruction, meshes can serve as extensions or substitutes for the pectoralis major, helping cover the implant and creating a fuller and more natural inframammary fold [24].

We placed implants prepectorally in cases without axillary lymph node dissection, whereas implants were placed subpectorally in other cases. Overall, 17 and 18 prepectoral implant and subpectoral implant reconstruction cases, respectively, were conducted in the endoscopic group, compared with 14 and 26 cases, respectively, in the open surgery group. This may have affected the BREAST‐Q scores. Additionally, we used smooth round silicone gel implants, which are more prone to displacement and capsular contracture than textured round or teardrop‐shaped implants. For patients with larger or significantly ptotic breasts, reconstruction outcomes may be suboptimal [25].

In conclusion, E‐NSM represents a potential improvement that seeks to achieve a pivotal balance among oncological radicality, aesthetic outcomes, procedural efficiency, and technical reproducibility. This evolution signifies a paradigm shift in breast surgical philosophy, moving from the principle of “maximal tolerable resection” to “minimal effective damage coupled with optimal quality of life,” offering increased options to breast cancer patients worldwide.

## Author Contributions

**Yuan Shao:** conceptualization, data curation, writing – original draft. **Youcheng Zhang:** conceptualization, writing – review and editing. **Huafeng Kang:** conceptualization, writing – review and editing. **Qing Zhou:** data curation, formal analysis, investigation. **Ziliang Zhang:** data curation, formal analysis, investigation. **Guiru Yan:** formal analysis, resources. **Binghua Kan:** funding acquisition, project administration, writing – original draft.

## Funding

This study was partially funded by the Scientific Research Project of the Health Commission of Shaanxi Province (Grant 2021D017).

## Ethics Statement

The study protocol was approved by the Institutional Review Board of Hanzhong Central Hospital (IRB#202424).

## Consent

Informed consent was obtained from all individual participants included in the study.

## Conflicts of Interest

The authors declare no conflicts of interest.

## Supporting information


Supporting Information S1

## Data Availability

The data that support the findings of this study are available from the corresponding author upon reasonable request.
