# Supplementary material for: Endoscope‐Assisted Nipple‐Sparing Mastectomy With Immediate Implant Reconstruction: A Retrospective Study
Source: World J Surg. 2026 Mar 20;50(5):1152–8. doi: 10.1002/wjs.70316 (PMC13206543; doi:10.1002/wjs.70316)
Supplement: Supplementary file 1 — Supporting Information S1 [file WJS-50-1152-s001.docx]

*World Journal of Surgery*

**Online Resource 1**

**Endoscopic-Assisted Nipple-Sparing Mastectomy with Immediate Implant Reconstruction: A Retrospective Study**

Yuan Shao^1^, Youcheng Zhang^2^, Huafeng Kang^3^, Qing Zhou^1^, Ziliang Zhang^1^, Guiru Yan^1^, Binghua Kan^1,*^

^1^Department of Surgical Oncology, Hanzhong Central Hospital, Shaanxi, Hanzhong 723000, China

^2^General Surgery Department, Second Hospital of Lanzhou University, Gansu, Lanzhou 730030, China

^3^Breast Disease Diagnosis and Treatment Center, Second Affiliated Hospital of Xi'an Jiaotong University, Shaanxi, Xi'an 710000, China

***Corresponding author:** Binghua Kan

Department of Surgical Oncology, Hanzhong Central Hospital

Shaanxi, Hanzhong 723000, China

Phone: 18009166001

Email: [kan0784@163.com](mailto:kan0784@163.com)

ORCID: 0009-0001-2027-7619

**Supplementary Methods**

***Criteria for Surgical Grouping:*** The rationale lies behind the fact that the open surgical technique for implant reconstruction embodies a classic, versatile, and robust option. Therefore, in patients with large tumor volume (diameter exceeding 5 cm), multifocal lesions, tumors located close to the skin, or those exhibiting significant breast ptosis, skin laxity, or thin subcutaneous tissue, open breast reconstruction with implants is generally recommended. These patients should be prioritized for the open surgical technique. In other scenarios, endoscopic surgery is the more preferred approach.

***Open Surgery Group:*** Patients were placed supine with the affected side elevated. Surgical incisions were made in any of these three positions: around the areola (periareolar), in a lateral radial pattern, or along the inframammary folds. Subcutaneous glandular resection was performed, and the nipple-areola complex was preserved. The pectoral fascia is typically preserved if both preoperative imaging and intraoperative exploration confirm the presence of a safe distance between the tumor and the pectoral fascia. The decision to preserve the pectoral fascia is typically made when preoperative breast magnetic resonance imaging (MRI) or mammography confirms a clear fat plane between the tumor and the fascia, with no radiological signs of invasion (such as fascial thickening, irregularity, or edema). This imaging finding is considered indicative of a safe distance from the chest wall, allowing for preservation during surgery. When a deep-seated breast tumor invades or is adjacent to the pectoral fascia, the involved fascia is routinely resected to ensure negative surgical margins. Intraoperative frozen section analysis was performed to evaluate the pathological status of the deep margins of the nipple–areolar complex. If the initial margin was positive, additional resection of the positive margin and excision of the major lactiferous ducts at the nipple base were performed. Complete nipple excision was performed if the additional margin remained positive [1]. The implant was inserted into the surgical cavity via an incision, and silicone drainage tubes were placed in the chest wall and axilla.

***Endoscopic Group:*** The tumor's surface projection on the breast was marked preoperatively. The surface projection of the sentinel lymph node in the axilla was identified and marked through ultrasound contrast imaging. A curved incision of 3–5 cm in length was made along the axillary skin crease. The incision was positioned at the apex of the axilla, posterior to the lateral border of the pectoralis major. Patients were positioned in the "looking at the moon" position (Supplementary Figure S1) [2], and the procedure was performed in accordance with *Expert Consensus and Technical Guidelines for Endoscopic Breast Surgery (2021 edition)* [3]. A wound protector and trocar were inserted through the axillary incision to establish an insufflated cavity. CO_2_ insufflation pressure was maintained at 10–13 mmHg. The retromammary space and ligaments of the glandular-fatty layer were dissected retrogradely through an endoscopic electrocautery hook. Dissection was performed medially to the parasternal border, laterally to the anterior axillary line, inferiorly to the triangular converging fascia, and superiorly to the upper pole of the breast, with careful preservation of the subcutaneous adipose layer. The entire mammary gland was excised en bloc (Supplementary Figures S2–S4), and the anterior fascia of the pectoralis major was preserved [4]. The remainder of the surgical procedure was the same as that of the open surgery.

***Inclusion and Exclusion Criteria***

Inclusion Criteria: Eligibility required histopathologically confirmed stage I–II ductal carcinoma (ductal carcinoma in situ/invasive ductal carcinoma per American Joint Committee on Cancer [8th ed.]), including pathological downstaging cases (ypT0-2N0) post-neoadjuvant therapy. Anatomical prerequisites comprised dual imaging-confirmed absence of nipple/skin involvement (MRI Breast Imaging Reporting and Data System 4/5 + mammographic correlation) and unifocal disease on preoperative MRI. Mastectomy indications included multicentric microcalcifications (≥3 foci on tomosynthesis) or extensive intraductal components (>40%) in breast volume <800 cc (Volpara V3/V4), contraindicating breast conservation. Additional criteria included reconstruction intent (BREAST-Q Desire ≥70), age 18–70 years (stratified by menopausal status), Eastern Cooperative Oncology Group 0–2, and American Society of Anesthesiologists class ≤III.​

Exclusion Criteria: Major cardiopulmonary dysfunction (New York Heart Association Class III/IV heart failure; left ventricular ejection fraction <45%; forced expiratory volume in 1 second <50% predicted); active gestation (β-human chorionic gonadotropin >5 mIU/mL) or lactation ≤6 months postpartum; prior/concurrent malignancies (excluding non-melanoma skin cancers) within 5 years per Response Evaluation Criteria in Solid Tumors v1.1; chronic corticosteroid use (>10 mg prednisone-equivalent/day ≥4 weeks) or post-transplant immunosuppression (calcineurin inhibitor trough >5 ng/mL); history of oncoplastic procedures (reduction mammoplasty/augmentation) altering breast anatomy; positive resection margins (≤1 mm) or pathologically confirmed residual malignancy (ypTis/T1mic+); and protocol non-compliance (>2 missed follow-ups or incomplete patient-reported outcomes).

***Postoperative Management***

Intravenous antibiotics were administered within 24 h post-surgery to prevent infection. Oral antibiotics were continued for 1 week. After awakening from general anesthesia, patients adopted a semi-recumbent position to facilitate breast reconstruction shaping. The negative-pressure drainage system pressure was maintained at a low level to prevent the skin flap around the implant from adhering tightly to the chest wall; adherence can expose the contours of the implant (Supplementary Figure S5). At 1 week post-surgery, patients were instructed to wear an elastic bra designed for breast shaping. Gentle flexion and extension movements of the affected side limb were permitted; however, actions such as raising or abducting the arm were restricted. Drainage was kept unobstructed. If the reconstructed breast appeared overly full or felt swollen, the negative-pressure drainage volume was increased appropriately. The drainage tube was removed when the daily drainage volume remained below 30 mL for 3 consecutive days. Approximately 3 weeks post-surgery, patients could begin gradual functional rehabilitation exercises for the affected side limb. After the drainage tube was removed, patients continued wearing the elastic bra for breast shaping for 3 months, during which strenuous activities were avoided (Supplementary Figure S6). Postoperative adjuvant treatments, including radiotherapy, targeted therapy, endocrine therapy, and chemotherapy, were selected based on pathological results.

***Observational Indicators***

The perioperative indicators were compared between the two groups. Analysis included: intraoperative blood loss, operative time, total drainage volume from the drainage tube, and total incision length. Total incision length was calculated as the sum of the axillary and breast incisions. Complications in both groups were compared and analyzed using Clavien-Dindo classification (CDC) and categorized as minor (CDC I and II) and major (CDC III and V) [5]. Aesthetics and patients’ quality of life were assessed at 6 months postoperatively. All patients completed postoperative questionnaires (Breast-Q and Scar-Q). Aesthetics was evaluated using the Harris scale [6].

***Postoperative Follow-Up***

Postoperative follow-ups, conducted every 3 months, comprised physical examinations, tumor marker testing, and imaging studies. The follow-up period ended in June 2024.

**References**

[1] Wang Y, Zhang B, Guan S, Zhang KT (2022) Incision selection for nipple-sparing mastectomy with implant-based breast reconstruction. Zhonghua Wai Ke Za Zhi 60:244–248. <https://doi.org/10.3760/cma.j.cn112139-20211202-00576>

[2] Tan F, Zhu Y, Luo Y, Xu J, Zhang P (2021) Application of position of “looking at the moon” in endoscopic surgery for breast cancer. J Pract Med 37:110–114

[3] Breast Expert Committee of the Minimally Invasive Division, Chinese Medical Doctor Association (2021) Expert consensus and technical guidelines for endoscopic breast surgery. Chin J Minim Invas Surg, 2021 edn. 21:1057–1067

[4] Zhang S, Xie Y, Liang F, et al (2022) Video-assisted transaxillary nipple-sparing mastectomy and immediate implant-based breast reconstruction: A novel and promising method. Aesthet Plast Surg 46:91–98. <https://doi.org/10.1007/s00266-021-02527-6>

[5] Dindo D, Demartines N, Clavien PA (2004) Classification of surgical complications: A new proposal with evaluation in a cohort of 6336 patients and results of a survey. Ann Surg 240:205–213. <https://doi.org/10.1097/01.sla.0000133083.54934.ae>

[6] Harris JR, Levene MB, Svensson G, Hellman S (1979) Analysis of cosmetic results following primary radiation therapy for stages I and II carcinoma of the breast. Int J Radiat Oncol Biol Phys 5:257–261. <https://doi.org/10.1016/0360-3016(79)90729-6>

**Supplementary tables**

Supplementary Table S1 Comparison of the operative time, intraoperative blood loss, total drainage volume, and total incision length between the two groups

| Variable | Endoscopic Group | Open Surgery Group | *t* | *P* |
| --- | --- | --- | --- | --- |
| Operative Time (min) | 198.71±31.82 | 123.50±17.77 | 12.844 | <0.001 |
| Intraoperative Blood Loss (mL) | 34.57±10.03 | 30.63±11.45 | 1.578 | 0.119 |
| Total Drainage Volume (mL) | 294.00±83.78 | 277.25±67.79 | 0.957 | 0.342 |
| Total Incision Length (cm) | 6.07±2.18 | 14.80±1.26 | -21.554 | <0.001 |

Supplementary Table S2 Comparison of postoperative complications and follow-up outcomes between the two groups

| Variable | Endoscopic Group | Open Surgery Group | *χ^2^* | *P* |
| --- | --- | --- | --- | --- |
| Minor Complications (CDC I-II) | 6 | 8 | 0.10 | 0.751 |
| Incision Infection | 2 | 3 |  |  |
| Implant Infection | 2 | 3 |  |  |
| Transient Nipple-Areola Complex Ischemia | 2 | 2 |  |  |
| Major Complications (CDC III-V) | 3 | 2 | 0.02 | 0.877 |
| Skin Necrosis | 2 | 1 |  |  |
| Implant Removal | 1 | 1 |  |  |
| Local Recurrence | 1 | 2 | 0.00 | 1.00 |
| Distant Recurrence | 2 | 3 | 0.00 | 1.00 |

CDC, Centers for Disease Control and Prevention

Supplementary Table S3 Comparison of Breast-Q, Scar-Q, and Harris Scale scores at 6 months postoperatively between the two groups

| Variable | Endoscopic Group | Open Surgery Group | *χ^2^*/*t* | *P* |
| --- | --- | --- | --- | --- |
| Breast-Q Score |  |  |  |  |
| Satisfaction with Breasts | 64.69±14.84 | 61.55±14.34 | 0.930^a^ | 0.356 |
| Physical Well-being: Chest | 69.20±15.55 | 67.20±16.09 | 0.545 ^a^ | 0.587 |
| Psychosocial Well-being | 74.97±14.92 | 71.85±16.85 | 0.844 ^a^ | 0.402 |
| Scar-Q Score | 76.11±15.21 | 64.55±19.98 | 2.789 ^a^ | 0.007 |
| Harris Cosmetic Score |  |  | 1.482^b^ | 0.756 |
| Excellent | 22 | 21 |  |  |
| Good | 8 | 10 |  |  |
| Fair | 4 | 8 |  |  |
| Poor | 1 | 1 |  |  |

Note: ^a^ indicates t-test; ^b^ indicates *χ^2^* test; *BREAST-Q^®^* *version 2.0 © Memorial Sloan Kettering Cancer Center and The University of British Columbia, 2017.*

**Supplementary Figures**

**
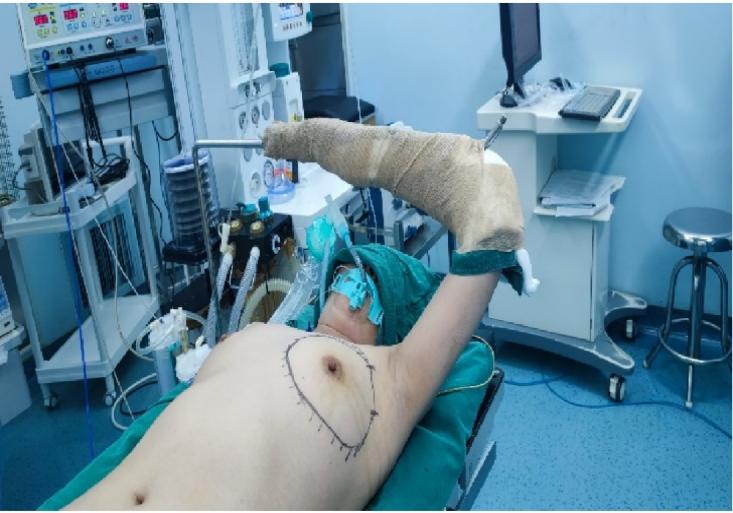
**

**Supplementary Figure S1** "Looking at the Moon" position


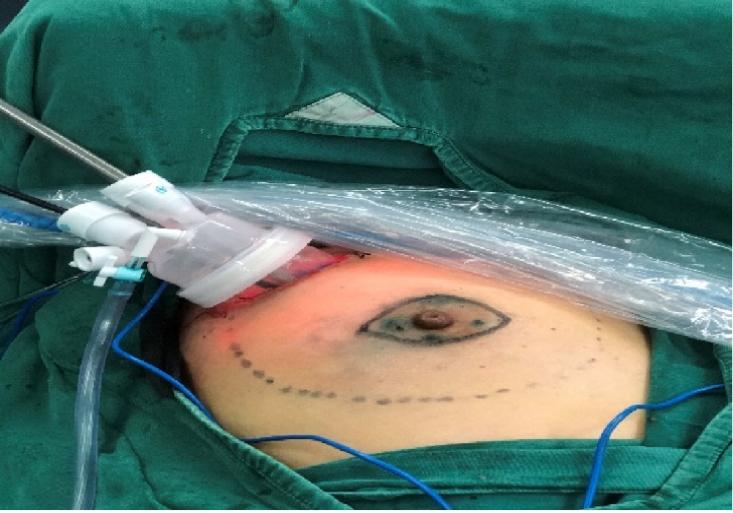


**Supplementary Figure S2** Surgical access port


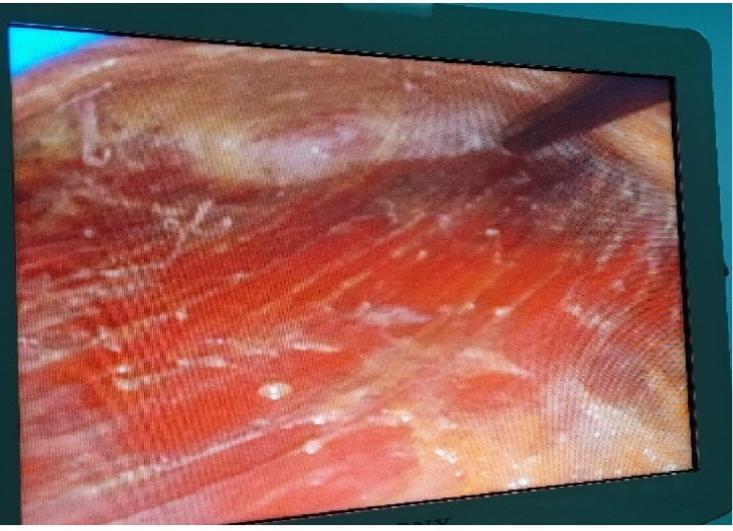


**Supplementary Figure S3** Retrograde dissection of glandular tissue


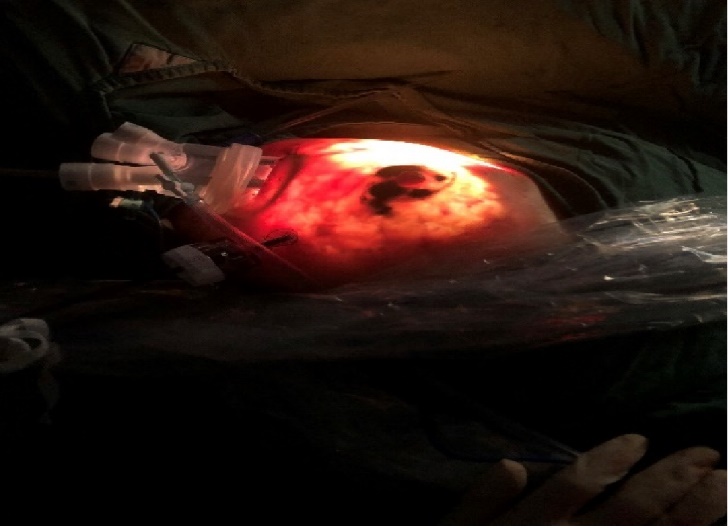


**Supplementary Figure S4** Post-mastectomy appearance resembling a "red lantern"


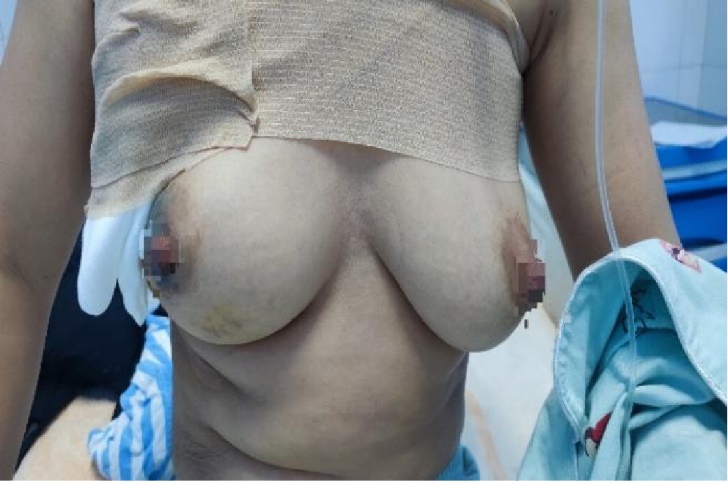


**Supplementary Figure S5** Postoperative elastic bandage fixation of the breast


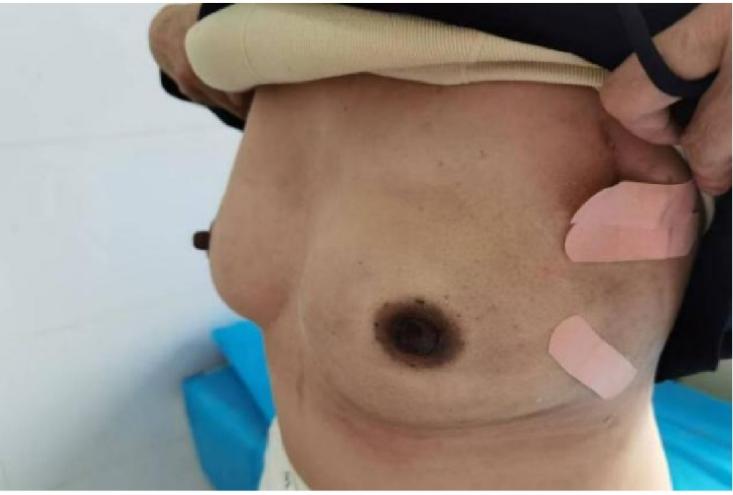


**Supplementary Figure S6** Small and concealed incision
